# American Dental Association

**Published:** 1896-03

**Authors:** 


### Reports of Society Meetings.

### AMERICAN DENTAL ASSOCIATION.

(Continued from page 41.)

*August 8, 1895.—Third Day.—Morning Session.*

Meeting called to order by Dr. Watkins, who presided.

The minutes of the previous meeting were read and approved.

Dr. Ambler's paper, "Titles and Number of Articles which have appeared in Dental Journals during the Past Year pertaining to Operative Dentistry," was read by title only.

The president appointed on the committee as to the condition of the mouth and teeth as bearing on life insurance the following gentlemen:

Drs. Frank Abbott, C. S. Stockton, J. S. Marshall, T. W. Brophy, and S. G. Perry.

*Dr. Taft.*—Dr. Corydon Palmer, one of the oldest practitioners in the country, is present with us to-day, and he would be pleased to have a few minutes to speak to this Association. I move that he be allowed twenty minutes, at some proper time, to address this society on any subject he may choose.

Motion carried.

*Dr. Crouse.*—Dr. John Tomes died about a week ago, and I would move that we take some action on his death.

Referred to the Committee on Necrology.

*Dr. Corydon Palmer.*—In what I have to say, I must refer to the past briefly. I see around me the older members of the profession, and also some new ones, whom I greet. It is well known to the older members of the Association that I began early in 1839 with the first meetings that were held. We had our great leader then, and I stood by his side and helped to bring forward his fine teachings.

What I have to say and to show is something which does not need to go into discussion. I only wish to announce it, and let you say whether it is good. Of late I have divided my time most concentratedly upon one appliance. I refer to an angle appliance for the hand-piece. There are a great many of them, and I have an admiration for them all. There has been only one kind of an angle that has been entirely overlooked, and I have endeavored through manufacturers and others to have it produced, but they say it cannot be done. The one I propose is a quarter angle, and what I wish to have you understand is that there is something about a quarter angle that is strong, and it can take the place largely of the straight hand-piece for excavating. It is my first effort, and entirely my own design. No one has yet looked upon it, and I place it first before you.

(Illustrating on black-board.) It will be found to be more convenient than a straight hand-piece. I do not say it is perfect. I have not fine engine machinery to do it with, and I was obliged to do it in a different way. I have something to say in reference to burs to be used in the hand-piece; it might be said I am pleading for trade and for manufacturers, but this body understands that I have never put anything on sale. What I have had I have given freely, and I have done it because I love my profession. This is the form I advocate; instead of making it as large in proportion to the bur, each of a different size, they should reduce this portion of the larger burs, and give us the advantage of all this part here to cut deeply into the tooth. (Illustrating.) On one-half of this card I have some burs of my own design that have never been shown, and on the other side are some of the other burs, which have been reduced to the size which I consider best. What we want is a wheel bur, the shank part of which is reduced to a small size and long enough to give us the advantage of all that the bur is worth. My burs cut with a drawing stroke; ordinarily they are not made that way.

There is a great deal being said about microbes and leucocytes. I have nothing to do with them. I have a drill here to give the final touch in the foramen of a tooth. The ones we have had are so formed that they carry before them the devitalized and putrescent matter which a root contains, and push it through the point of the root. I have endeavored in making this nerve instrument to have something which will take away with it that portion which it cuts, and not carry it before it. Here is a little thing which I want to show you, to follow that delicate portion to the end of the root. It will bring away with it, to the very last touch, all that it has cut off. I would like to have some steel for these instruments which has a tough fibre. If we could get piano wire large enough to hold, it would be a good thing.

The following is the discussion of the papers read last evening by Drs. Smith and Patterson:

*Dr. Smith.*—I purposely present myself this morning for the opening of this discussion that we may discuss the paper which was read fairly and from the right stand-point. It seemed to me last evening that there was a disposition to misunderstand the intent of the paper. The first gentleman spoke of the kind intentions of our heavenly Father. He said, if it was the design of Nature that the tooth later in life should present itself in such a way that the pulp should be of no value, then Nature would have made provision for taking away the pulp. The intention of the paper was that we might better understand not the destruction, but the preservation of the pulp, and to place it and the teeth, which it protects, in its right light. It was not the intention of a kind Providence that we should see men on crutches or with empty sleeves, but we do see them; and we see another thing,—that He left to the human race the solving of the problem how best this condition of things may be met, just as He has left to the human race the problem of how best to meet the disabled conditions of the human teeth. It is for us to work it out. We are to do it in the right spirit. The pulp of a tooth is not a holy thing. We may operate on it if we find it necessary to do so. It is the teaching of this paper that wherever the pulp stands in the way of making the tooth useful we are to destroy it if necessary. The intent of the paper was not that there should be a wholesale destruction of pulps, but whether we are to regard it in young life or in middle life and old age as so sacred that we are not to approach its domain. The teachings of the present and the past have been without discrimination in regard to the dental pulp. There is a period of life when it is a holy thing. It is in the holy of holies, and is not to be interfered with; but later we may enter the domain of the pulp with perfect assurance, when it stands in the way of our doing the best we can for the relief of the patient, and then it is for us to destroy it.

In reply to Dr. McKellops: I estimate the matter of education among the masses of such importance that I would gladly receive from this Association any suggestions in relation to the matter. I think it is the best thing for us to do for the masses who are suffering. I would be very glad if this Association would discuss the point, and do something for the relief of the poor. The impression seemed to be given us yesterday that even the dental colleges should be suppressed, and the clinics and infirmaries suppressed rather than widened. However many there are, however great the number of students, they do not begin to touch the number of suffering people who should have relief.

My friend Dr. Truman turned the sentiments of the paper into a channel entirely foreign to the plain meaning thereof, and because he said, after having done that, "I do not believe it," he did not change the facts that are there.

I have a reply to the discussion as it was entered into by my good friend Dr. Black. I have the profoundest regard for Dr. Black. I have read his papers and watched his course, which is in the interests of the dental profession. It may be twenty years ago that I saw a little article in one of our journals by Dr. Black, and I have watched him from that time. He gave me in that little article a hint which has been of the greatest value to me. He said, "Why is it that some days your gold-foil does not work well, and on other occasions it seems to work very well, all of it being from the same book? If you look to your annealing-lamp, possibly you will find the burnt end of a match lying on the wick, and this is the whole secret, because of the phosphorus which is emitted from the end of this burning match, which interferes with the working of the gold." I do not know that I ever light my spirit lamp without thinking of Dr. Black. A man who will throw out such hints as that to the profession is a man to be respected, and whose opinions are to be regarded.

There was nothing in the whole discussion last evening, or in the preparation of the paper, that so pleased me as did the remarks of Dr. Black. Although seeming to be in entire opposition to the paper, they were in entire confirmation of it. He stated that this consolidation of the tooth which we speak of, and to which we attach so much importance, began in early life, that it continued to the period of about fifteen years, when it lessened, to a later period, when it again increased, and in middle life it almost seemed to stop. Is not that almost the same idea that I mentioned in my paper? He said there was a change, and that is what I claim. There is a change in the tooth-structure. Destroy the pulp at six or eight or ten years of age, and you have made a tooth which yields readily to decay. Keep it in good condition to middle life, and you have so changed it that it has become decay-resisting. The vital forces within the tooth have fought against the externals, which have been attacking it, and they have conquered, and built the tooth into a material which is decay-resisting.

The paper was entitled "The Office of the Dental Pulp and its Eccentricities." I intended that that should be one of the eccentricities of the pulp,—not a normal condition. The tooth which he analyzed may have been one which had really been consolidated, but I do not claim that that is the normal condition of the pulp. There are instances where the tooth actually becomes softened. Whether it is due to an increase of density in the tooth or to some other cause I care not. No man will dare to say that these teeth do not change and become soft, and fillings that have preserved cavities for years begin to drop out through softening around the cavity. I claim it is the action of the pulp that does it.

*Dr. Abbott.*—From the way Dr. Smith's paper read last night, and the remarks of Dr. Black following it, you would certainly judge that they were in opposition to each other. The paper stated most positively that there is a change in a tooth in old age, which apparently would indicate decalcification of the dentine. I think this is true, but I have never attributed it to the action of the pulp. I believe the pulp keeps on depositing lime salts as long as the pulp is alive, and the tooth becomes more solid and perfect every day. Eventually, even the pulp-chamber itself becomes obliterated. I have never seen a case of softening anywhere excepting when the necks of the teeth and quite a portion of the roots had been exposed. I have always attributed this condition to the fact that there was an acid that was slowly decalcifying the tooth; that there was an action that was absolutely pathological. I may be wrong, but I am positive that the action of the pulp is going on in ordinary teeth constantly in the form of supplying lime salts.

The question of pyorrhœa came up in the paper as read last night. It was asked, "Why is it that teeth that do not decay and are the best we find in the mouth, seem to be more subject to this deposit than those which have to be cared for or are filled?" I think it is due to the fact that the persons whose teeth decay constantly have visited a conscientious dentist and had the tartar removed from them. The result has been that in one instance every particle of tartar is removed from time to time, and in the other it is not. There are people who will boast that they have never been in a dentist's chair.

In reference to the second paper read, I want to speak of the materials used for capping the pulps of teeth. I am in favor of saving them always and in every instance. I consider it a crime to destroy the pulp in a tooth. We want the material for exposed pulps that will save them and keep them comfortable for a long time. It was recommended in this paper that gold be used by placing it over the cavity and burnishing it. If any space is left between the exposed point of a pulp and the material you place over it, that space will fill with serum, notwithstanding you may use escharotics, and you cannot by any means place gold or any metal there that will not admit it somewhere.

*Dr. Patterson.*—I did not advocate any such practice. I was referring to deep-seated cavities, not near the pulp.

*Dr. Abbott.*—Then I have been mistaken. But the point is a good one nevertheless, and I want it understood. The material that is necessary for capping pulps of teeth must not expand nor contract and it must fit the opening perfectly, and these qualities must be present with every case or we will fail. Another point in the treatment of a pulp is that the pulp should never be punctured by any means or it will die. Gutta-percha was recommended for capping the pulps of teeth. This fails with me invariably, and the pulp will die, because gutta-percha expands and it presses upon the pulp, and the slightest irritation—the weight of a hair, even—will cause the death of the pulp eventually. Usually I recommend that this be not used with any idea of success in the capping of pulps of teeth.

*Dr. Shields.*—From Dr. Smith's reply this morning, I took it for granted that he was under the impression that the discussion was closed. A very important point in his paper has not been reached. He said, after a pulp has been saved for a number of years, the cavity can be filled with gold, and owing to the condition of the pulp and the change in the dentine, you will find around the margin of the tooth blackness, which is nothing but defective workmanship, and the dentine has nothing in the world to do with it. The question of the compatibility and incompatibility of gold should not be entered into. If the gold is properly introduced into a cavity, you will never have a black rim around a filling.

*Dr. Rhein.*—The point in Dr. Smith's paper, in which he refers to the care of children's teeth, is one to which we should give our attention. If there is any body of dentists in this country, in an official capacity, which owes a duty to the public, it is this Association. That point of the paper has struck a responsive chord within me. I believe that it ought to be part of our duty to endeavor to do something in the way of improving the teeth of future generations in this country. I refer to the condition of children's teeth before they are admitted to the public schools for the daily session. If, as a national organization, we would send some communication to the various boards of school commissioners throughout this country, informing them of the value of examination of children's teeth before entering upon their studies, one of the greatest benefits for the future condition of the teeth of the people of this country would be achieved.

A great deal of stress is placed in the public schools upon the general condition of the cleanliness of the scholars when they make their daily appearance. I believe the children are not admitted to the sessions with dirty hands or faces, but are sent home to be washed. We know very well how much greater importance ought to be placed on the condition of those children's mouths.

In reference to the condition of pulps, I wish to say a word. I agree with Dr. Smith and the other gentlemen in regard to the preservation of the pulps of teeth before they have reached the age of maturity, before the tooth-structure has been properly consolidated, and I agree with the distinction the author of the paper has made, that the class of cases to which he had reference were not normal physiological conditions, but conditions that were pathological. There is some disturbance of the nutrition, usually a lack of nutrition. Professor Black referred to this point in his discussion last night. It is nutrition either in excess through excessive irritation, or a lack of the proper protoplasmic irritation. Is it advisable to allow an adult tooth to go on with a condition of abnormality about the pulp, or is it better to remove such a pulp? The weight of all the experience which I have had on this subject has been towards the immediate removal of a pulp as soon as an abnormal condition presents itself. Many of the gentlemen to whom I have listened, and who advocate the preservation of the pulp of a tooth at all hazards, are not aware of the fact that these cases may find themselves in the offices of other men, with abscesses or other conditions due to the fact that they have endeavored to save an organ whose usefulness has departed.

I do not agree with the author in his remarks on pyorrhœa alveolaris. In his very admirable paper he does not differentiate between what forms of pyorrhœa he has reference to. There has been under observation a very limited class of pyorrhœa cases which have their origin in pulp disturbances and make themselves known around the apex of the root; but these cases are very rare, and constitute but an infinitesimal portion of the pyorrhœa cases that present themselves. I want to take issue with the statement that pyorrhœa does not present itself in youthful patients. In the paper on "Infantile Scorbutus," presented before the First District Dental Society of New York, by Dr. Kirk, and published in the *Dental Cosmos*, he distinctly calls attention to infantile pyorrhœa, and I referred to a similar case that reached my hands. The mother of the child had been formerly under the attention of Dr. McManus, of Hartford. The child was eighteen months of age at the time. I have noticed pyorrhœal cases in children of all ages after various diseases, such as typhoid fever, scarlatina, and diphtheria,—diseases which interfere with the nutrition of the body to a great extent. But it is probable that these conditions do not reach the notice of the dentist unless they are looked for. The dental organs are in a much more healthy condition at that age than we find them later in life. After the patient has recovered from such an illness, it takes but a few weeks to get back to a healthy condition. If the gentlemen will visit the wards of the hospitals where there are cases of this kind, I challenge their observation of the gums of such patients, especially in the convalescent stages of disease. I lay special stress on this point, as I did last year, because it is time that we should distinguish what form of pyorrhœa we are discussing. With reference to the limited class of pyorrhœa which the author of that paper speaks of, I agree with him, but I wish to have that discrimination made because it refers to only a very limited number of such cases.

*Dr. McManus.*—I wish to say a word or two about that case, because a gentleman asked me why I neglected my patient and had to have the case go into the hands of Dr. Rhein. The patient he speaks of is the child of a patient of mine. She was my patient before she married for a number of years; she married a physician in New York, and this little child of hers needed the services of a dentist. She wrote to me asking to whom she should go; I recommended Dr. Rhein, and they have been very much pleased with Dr. Rhein's services.

*Dr. Hunt* (of Iowa).—I followed the reading of Dr. Smith's paper with considerable care, but I was confused in the terms that he used that were not familiar to me in the connection in which he used them. I hope before the discussion of the paper is closed he will make some explanation. In referring to the dentine and enamel, he used the terms "rebuilding" and "recalcification." He also speaks of a pulpless tooth. I think the statement is that pyorrhœa does not attack a tooth in which the pulp is destroyed. Three or four years ago, Dr. Miller, of Iowa, announced that pulpless teeth were not affected by pyorrhœa, and teeth adjoining pulpless teeth yielded to treatment rather more readily. I have given some attention to this feature of pyorrhœa, as to whether there was any difference perceptible in the condition of the tooth,—whether more amenable to treatment or not, if the pulp were devitalized. I do not as yet observe anything that would warrant me in thinking that there is. However, this difference must be demonstrated in some other way than by clinical observation. Another point in the paper which seems very strange to me is that the doctor should claim that the pulp becomes in old life an element of destruction of the teeth. The indications for this are that teeth begin to soften around fillings in a good class of teeth. I have always supposed that destruction of the teeth, or caries, as it is commonly called, must proceed from without. I supposed that this had been demonstrated quite clearly. I think the condition he mentions can fairly be accounted for upon that fact, that heretofore all of the conditions of the disintegration of the teeth have been caused from exterior conditions. We shall have to look a little further than the ordinary dental operation to find the cause.

It is very common among people past middle life to be afflicted with various diseases; hardly any one past that time of life is entirely free from some disturbance, either of the vital organs themselves or of the parts in close connection with them. My own opinion is that this condition of the softening about the margins of fillings is due to a changed condition in the mouth that presents a more favorable field for bacteria. Nearly all of the disturbances of the kidneys, liver, lungs, etc., would account for this change. In one case—that of a gentleman of fifty-five years—I found this same condition in the mouth. I was curious to know what caused it, and I removed a portion of the decay and sent it to the pathological laboratory for examination. The report was that the individual was afflicted with pulmonary phthisis. I did not expect that at all, because there were no external signs of it; but I thought there was a constitutional disturbance which caused certain secretions. We will have to change our views about what causes decay in teeth if it is demonstrated that the pulp becomes an organ of disintegration. We have an abundance of proof that this organ is constantly carrying nutritive material and consolidating the teeth.

In regard to a point in Dr. Patterson's paper, I think the statement was that non-cohesive or soft foil could not be made cohesive,—in other words, welded together,—and because of this fact, the soft gold became a better non-conductor of thermal changes. One instance came to my mind. Not very long back, Dr. Gardiner had occasion to examine a tooth filled by Dr. Allport thirty-five years before. The tooth had become so badly disintegrated that it was necessary to remove the filling, and Dr. Gardiner out of curiosity wanted to know what kind of gold was used in that cavity. Dr. Allport, who kept a register, reported that it was filled with soft foil. It was passed through a set of rolling-mills and rolled into a sheet. If there had been no welding of the sheets of the foil, that could not have been done.

Dr. Jack moved that, inasmuch as there were so many papers, the discussion be limited to five minutes for each speaker. Carried.

Dr. Stainton moved that the gentlemen who read the papers be given five minutes each to close the discussion. Carried.

*Dr. Smith.*—I had hoped that you would allow me to say just a word about this point: as to whether there was any difference between the devitalization of the pulps at different periods of life. Dr. Abbott claims there is not, but it seems to me there is. Pulp-capping has been alluded to, and the question asked at what period shall we do our pulp-capping. The point of the paper is this: that if a tooth has resolved itself into a pathological condition of the exposure of the pulp, or any other reason, the pulp can be destroyed, and it is better to do it than to cap the pulp. It seems to be implied that it is just as easy to attempt the capping of a wisdom-tooth on its distal face, or the distal face of an upper or lower molar decayed way down to the root, as to cap any other tooth. Notwithstanding, all these distinctions should be carefully made. Who dare attempt to destroy the pulp of a lower wisdom-tooth exposed from the distal face? It is a risk in regard to the life of a patient. Who dare attempt the capping of a pulp in a lateral incisor where your room is limited? In a lower molar tooth, where the conditions are favorable, we can discuss the question of capping the pulp. It never entered my mind to cap the pulp of a bicuspid or lateral after the pulp has become consolidated, because of the almost certainty of the unfavorable results which would follow.

*Dr. Patterson.*—In regard to the non-cohesive gold, I want to say that it is not amenable to any welding process. The soft foil that Dr. Hunt referred to is soft cohesive foil, which is entirely different. The distinction is not very clear as to what dead, soft, non-cohesive gold is. There can be no welding between one sheet and the other, and there never is.

*Dr. Rhein.*—Before we leave the subject I would like to offer to the Association the following resolutions embracing the point of children's teeth, mentioned in Dr. Smith's paper:

*Resolved,* That a committee of three be appointed whose duty it shall be to draw up a suitable circular letter embracing the recommendation in Dr. Smith's paper concerning the cleanliness of teeth of school children. They shall cause that to be sent to the various boards of education throughout the United States, and to be sent to the dental journals and press generally.

*Resolved,* That such committee be reimbursed from the treasury of the Association for any expense they may incur.

Motion made to lay the resolutions on the table.

*Dr. Bryan* (of Switzerland).—I have been informed that you have done me the high honor of making me an honorary member of this society. I want to thank you, and to say that if I am not now worthy, my future efforts in dental life shall be to earn and deserve that honor.

Dr. Bryan also read a short paper on "The New Mat Gold," of which an abstract follows:

The dream of the dentist since the discovery of the cohesive properties of gold-foil has been to find a cohesive gold preparation which would be sufficiently plastic to be used as the cements and amalgams of the day are used. Within the last few years a limited circle of dentists in Switzerland have passed the word that Dr. de Trey had solved the riddle, and was in possession of the secret of preparing an ideal plastic gold. Within the last few months the gold has been placed on the market, and our Swiss friends are quite enthusiastic about it. If in time Solila gold does not satisfy our wants, and prove the ideal gold which many disinterested people of to-day think, it will certainly indicate the road to other pilgrims seeking the ideal, for it is a step in the right direction.

As so much was being said in praise of the Solila gold, I went to Dr. de Trey, despite certain personal differences, and asked him for some information about the gold, but I was unable to learn anything about the preparation of it. The doctor filled some teeth out of the mouth in a remarkably short space of time, and the manipulation was such that I had my doubts about the adaptation and general cohesion of the mass of gold condensed by hand-pressure and very slight malleting, building up a contour filling, with no undercuts or retaining points, in certainly one-quarter the time usually employed in such cases by the usual methods. I not only had the curiosity to have the filling removed and rolled out, but I wanted to see it done, and was taken into the laboratory, where the filling was rolled out into a beautiful yellow, soft ribbon of about No. 250, without a defect, as far as I could see.

Dr. Jenkins, who is now the president of the American Dental Society in Europe, reports much pleasure in its use for the short time it has been on the market, but says he prefers to finish the filling with a gold which does not require so much time to finish down, owing to its extreme hardness, even after hand-pressure condensation. This gold should not be malleted like other gold, as the first stroke condenses the point under the packer, and a second blow will cause pain and be of no advantage. Dr. Patterson reports very great facility in rapid filling, little loss of gold, and superiority to the other crystal mat golds on the market, but, like myself, can only judge of it superficially. For the last month I have used this gold exclusively, as a test of its general adaptability, and I am generally pleased with the results.

Dr. Bryan also showed several instruments which he had brought with him, including a sponge-holder with mirror, a mirror with shell-shaped rim for carrying fillings to teeth or parts of teeth that are not easily reached, a cervical clamp, and some matrices, together with a stand to hold the same.

Dr. Shields, of New York, read his paper, entitled "Scientific Filling of Difficult Roots." He said that all operative dentistry is minor surgery. All dental operations are the embodiment of mechanism of the highest order. Whenever we have to substitute any portion of our anatomy we at once undertake a most difficult task. We are fearfully and wonderfully made, and no branch of medicine so thoroughly recognizes this fact as dental surgery. The tooth becomes decayed from the inability of the dental pulp to throw out odontoblasts sufficiently fast, and it becomes exposed and dies. We are called upon to fill the tooth, and make it as perfect as it was before. Our Lord made our teeth, and poor finite man can only fill them imperfectly; but we must come as near perfection as we can. We must reach the apex of each canal, and we must not know the meaning of the word "fail." All canals can be thoroughly cleansed to their very apices, and not a particle of decomposition remain. Apply the rubber dam, and always open into a canal with a bur, because you will not block the mouth of the canal with large pieces of tooth-structure. Save all the tooth-structure possible, but do not allow tooth-structure to prevent your entrance into a canal. After opening into the canal with a bur use Donaldson's nerve-canal cleaners, Nos. 4 and 5. With a small cleanser and a little perseverance, I can readily reach the apex of a difficult canal. I puncture the small piece of rubber dam with the cleanser, and can reach the apex, and indicate with the dam the exact length of the root from apex to grinding or cutting surface. I clean the canal, and treat it with non-coagulating disinfectants. Then I introduce a larger cleanser, and if it should block up the canal, the obstruction can easily be removed by inserting the original cleanser, and turning it around a few times between thumb and forefinger. I use soft gold-foil No. 4, and if I am filling a small canal, do not fold it at all, but cut it into strips and the strips into pieces about one-quarter the size of a pin-head. The gold displaces the air in the canal, and gold being a solid substance is readily indicated by the indicator when it reaches the apex. I know I fill the apex. In surgery we do not guess at things, but we coolly calculate them, and know that they are correct.

Dr. Freeman then read a paper entitled, "The Use of Compressed Air in Operative Dentistry," and illustrated the same with numerous instruments. An abstract of the same follows:

Every workman in the exercise of his art should be provided with proper implements. The dentist who is called upon to treat and relieve pain of such delicate and sensitive structures as the teeth should naturally look forward to that which can assist him in the practice of his profession, and it is essential to his success that he be provided with all the necessary appliances which will aid him in his operations and mitigate the sufferings of his patients.

No procedure appears to me to be more conducive to success than the application of compressed air. Permit me to explain the methods of producing compressed air, and the manner of conducting the same to your operating-chair. It is obtained by means of a suction-pump, which soaks in the air and forces it through a pipe to a reservoir. I would not advise the use of a small hand-pump or cylinder, as you will find that both the air and the operator become quickly exhausted, but would recommend that which is known as the champion beer-pump, or the compound pump of the same manufacturer, as I find them the most satisfactory air-compressers on the market to-day.

Before placing the pump in your office ascertain how many pounds of water-pressure you have. If, as in my office, you have only twenty-five pounds, the champion pump will not furnish (contrary to the claim of the manufacturers) the same amount of air-pressure as water, as there is a loss of a few pounds. Such being the case, the compound pump is preferable, as it is frequently necessary to have the air at a pressure of forty to fifty pounds to the square inch. This pump we connected with the reservoir, which is a tank containing eighteen gallons of air, tested to one hundred and fifty pounds pressure to the square inch. From this runs a quarter-inch block-tin pipe, with branches to my laboratory and operating-room. To this I had attached a regulator and a gauge, the regulator being nothing more than a screw-valve; as you loosen the screw you close the valve, and *vice versa.* From the regulator a pipe leads to the gauge, which indicates the number of pounds pressure. To the gauge is connected a distributing-pipe, with three small cocks to attach the rubber tubes and the cut-off. The tubes should be of heavy rubber, so as to withstand a high air-pressure. The cut-offs close automatically, and you may use the somewhat antiquated expression, "Touch the button, and the air will do the rest!"

In the practice of dentistry it is absolutely necessary to have the mouth in an antiseptic condition. To produce this effect many of you use a hand-atomizer, which by the continuous pressure of the bulb causes a slight cramp in the hand. With the compressed-air apparatus, we do away with this work. You have the tube steadied, and can thoroughly cleanse the mouth or any cavity. I derive excellent results from the following prescription:

℞ Borine, 1 part;
Pyrozone med. 3 per cent., 2 parts;
Water, 1 part.

No doubt you have frequently met cases where you would prefer to place on the rubber dam, but owing to the patient's inability to breathe through the nostrils you were compelled to send your patient away or work at a great disadvantage. The remedy is a simple one. A two-per-cent. solution of cocaine placed in an atomizer attached to your compressed-air apparatus, which is gauged at ten to fifteen pounds pressure, and the spray thrown into the nostrils, will invariably relieve that posterior nasal catarrh, or reduce the swollen tissues of the nares to such an extent that you can proceed with your work in a few minutes.

In using the spray for this purpose you have your patient sitting upright in the chair, the head inclined slightly forward. Insert the tube horizontally into the nares, and do not apply over ten to fifteen pounds of air-pressure, as otherwise you may set up an irritation of the middle ear.

In stomatitis of the various kinds the spray, employed with a high pressure produces excellent results. Before applying any medicine to the gums it is always necessary to have a dry surface, so that the medicament may be readily absorbed, and not distribute itself over the surface of the tissue. I found it somewhat difficult to obtain a dry condition of the gums in the posterior portion of the mouth before employing this apparatus. Now I find it a very simple matter. By drawing back the corners of the mouth with a napkin or piece of cottonoid, and throwing the compressed air directly to the spot, it only requires a few seconds to procure the desired condition of the mouth, and upon applying your medicines you get immediate absorption.

In the treatment of pyorrhœa alveolaris it is absolutely essential that every particle of deposit, of whatever form, shall be removed, and the *débris* thoroughly washed away, leaving no particles to be ulcerated. To do this it requires a vigorous stream of water. Dr. J. N. Farrar introduced for this purpose a set of four syringes with delicate points. I use this attached to my compressed-air apparatus, at fifty to sixty pounds pressure, which throws a stream with such force as to entirely cleanse the parts. I employ these spray tubes with my compressed-air apparatus for introducing medicine and washing out cavities preparatory to implanting teeth, and I may be concise and say for all wounds and diseases of the oral cavity.

After considering the advantages derived from the aid of compressed air upon the soft tissues I will briefly state the results procured through the medium of these agents on the hard tissues,—the teeth.

It is not necessary for me to cite in detail the minute anatomy of the teeth, although a thorough knowledge of the structure of the dentine is requisite to fully appreciate the ideas which I wish to convey.

Drs. Harlan, Kirk, and Truman demonstrated by experiment that there is an absorption of liquids by osmotic action. Their statements have awakened the profession and provoked no little discussion, although the controversial period is usually only a passing, and never the most fruitful, period of any new truth. These gentlemen have established the fact that medicines will be absorbed through the tubuli after a certain length of time. You will remember that the glue-yielding portion of a tooth contains water to about ten per cent. of the weight of the entire tooth. Bearing this in mind, let me ask you what can we accomplish after dehydration of the tissue, leaving entirely out of consideration the capillary attraction of these infinitesimal dentinal tubes? It has been demonstrated that when water is removed from a tooth the normal function of transmitting impressions seems to be modified. We know that heat and cold will produce pain, and in the application of either we should proceed with extreme caution.

I use a hot-air syringe similar to the S. S. White No. 30, only it has twice as large a cylinder. The chamber is filled with carbon, which is one of the best materials for retaining caloric. With these syringes attached to the compressed-air apparatus, you can so regulate the flow of air that in from one to five minutes you have your tooth thoroughly dry. Then introduce your medicine, heated to about 95° F., and again applying your warm-air current, with about forty pounds pressure, you will be able to excavate your tooth without pain, nor will you have any subsequent irritation. In the bleaching of teeth, I find that by the application of hot air at a high pressure I am able to produce the required condition in one-half the usual time, as you rapidly evaporate the pyrozone twenty-five per cent. and force it into the tubuli.

For drying out root-canals use this small point on your syringe, and you can carry heated air directly into the canal, and with a high pressure can force your antiseptic through the canaliculi and tubules, rendering the tissues thoroughly aseptic.

In setting pivot teeth, we often find it difficult to carry the cement to the upper portion of the root. In forcing it into the root-canal with a dry instrument, upon withdrawal of the latter the cement is found adherent to it, not to the walls of the root; but with a high pressure you can force it into every irregularity of the root, while at the same time the compressed air will dry all the surrounding tissue, and you avoid the necessity of wiping away the moisture excreted by the mucous glands.

Dr. Taft moved that the Committee on the National Museum consist of five members instead of three, and the motion was carried.

Adjourned.

(To be continued.)

